# The importance of physician knowledge of autism spectrum disorder: results of a parent survey

**DOI:** 10.1186/1471-2431-7-37

**Published:** 2007-11-20

**Authors:** Rachel A Rhoades, Angela Scarpa, Brenda Salley

**Affiliations:** 1 Department of Psychology, Virginia Tech, Blacksburg, Virginia 24061, USA

## Abstract

**Background:**

Early diagnosis and referral to treatment prior to age 3–5 years improves the prognosis of children with Autism Spectrum Disorder (ASD). However, ASD is often not diagnosed until age 3–4 years, and medical providers may lack training to offer caregivers evidence-based treatment recommendations. This study tested hypotheses that 1) children with ASD would be diagnosed between ages 3–4 years (replicating prior work), 2) caregivers would receive little information beyond the diagnosis from their medical providers, and 3) caregivers would turn to other sources, outside of their local health care professionals, to learn more about ASD.

**Methods:**

146 ASD caregivers responded to an online survey that consisted of questions about demographics, the diagnostic process, sources of information/support, and the need and availability of local services for ASDs. Hypotheses were tested using descriptives, regression analyses, analyses of variance, and chi-squared.

**Results:**

The average age of diagnosis was 4 years, 10 months and the mode was 3 years. While approximately 40% of professionals gave additional information about ASD after diagnosis and 15–34% gave advice on medical/educational programs, only 6% referred to an autism specialist and 18% gave no further information. The diagnosis of Autism was made at earlier ages than Asperger's Disorder or PDD-NOS. Developmental pediatricians (relative to psychiatrists/primary care physicians, neurologists, and psychologists) were associated with the lowest age of diagnosis and were most likely to distribute additional information. Caregivers most often reported turning to the media (i.e., internet, books, videos), conferences, and other parents to learn more about ASD.

**Conclusion:**

The average age of ASD diagnosis (4 years, 10 months) was later than optimal if children are to receive the most benefit from early intervention. Most professionals gave caregivers further information about ASDs, especially developmental pediatricians, but a sizeable minority did not. This may reflect a lack of training in the wide range of behaviors that occur across the autism spectrum. Parents turned to outside sources to learn more about ASD. We recommend that all physicians receive specialized training about ASDs to improve upon early screening and diagnosis, and then advise caregivers about empirically-supported services.

## Background

Autism Spectrum Disorders (ASDs) are chronic and lifelong pervasive neurodevelopmental disorders that affect children's social, language/communication, and behavioral development. Impairments are evidenced by restricted social interaction and communication, and stereotypic/repetitive patterns of behavior or interests [1]. ASDs can severely impact when or if a child learns to speak, the development of social relationships, and the ability to integrate sensory information. Early diagnosis is vital for children with ASD and their families in order to facilitate earlier intervention, appropriate education planning, and the arrangement of family support services [2]. Delays in diagnosis cause delays in intervention which can then jeopardize prognosis [3]. After a child receives a diagnosis of ASD, it is critical that parents receive support and guidance about appropriate empirically supported services and treatment options. For these reasons, it is important to consider ways to improve the process of early diagnosis and referral for intervention.

One problem that can arise with this process is the late diagnosis of ASD. Research in the United States, for example, found that parents noticed symptoms as early as 6 months of age, but diagnosis was not until age 3 or 4 years [4]. Similarly, a study in the United Kingdom found that the average age of diagnosis of a child with ASD was 6 years, even though most families felt that something was wrong with their child at 18 months and sought medical attention when their child was 2 years old [2]. Others have found that the average delay between when a parent first seeks help and the time of diagnosis is 4 years [5,6]. It has been suggested that parents are often correct about concerns with their child's development [7], and symptoms of autism can frequently be seen before 12 months of age [8]. So, it is significant that families are often aware of atypical development long before a diagnosis is established.

Pediatricians and family practitioners are usually the first healthcare providers that a family contacts for children under 5 years old, which is the critical age for a diagnosis of ASD [9]. Some possible reasons for a delay in diagnosis are that professionals may be concerned about the strong emotional reaction of parents when they are told that their child has autism [10], fear of negative consequences from labeling the child, and hope that the symptoms will reverse [11]. Furthermore, some medical providers believe they have less adequate training than needed for assessing ASDs [12].

A second problem that can arise in the diagnostic process involves providing caregivers with accurate information about ASD and related treatment options at the time of diagnosis. A study in France found when parents express concerns about ASDs to pediatricians, the pediatricians often trivialized the disorders because of lack of knowledge about them [4]. Other research indicated that 4^th ^year medical students performed poorly on questions in a survey about the causation, IQ profiles, prognosis and treatment of autism [13]. Moreover, a survey of families of individuals diagnosed with ASD indicated that 33% of physicians spontaneously discussed non-traditional therapies for autism, which did not have strong empirical support [12]. These findings suggest that physicians may lack knowledge about certain characteristics and empirically supported treatments for ASDs. If this is the case, critical opportunities for education about the disorder and for referral to appropriate services may be missed.

Importantly, in the face of the diagnosis and subsequent lack of information, parents/caregivers may turn to other sources of information that may or may not be externally monitored for accuracy (e.g., internet). While potentially valuable, these sources may at times also provide misleading or even inaccurate information. In addition, the large amount of information from these sources may be overwhelming and confusing.

In order to assess the state of these issues, an online survey was conducted to assess the diagnostic process, available services, and current needs of individuals with ASD in Virginia. It was predicted that 1) consistent with Planch et al. [4], most children would be reported to be diagnosed around age 3–4 years old, 2) the majority of respondents would report receiving little to no information about ASDs from the diagnosing professional, and 3) the majority of respondents would report turning to other sources of information about ASD outside of their health care or service provider.

## Methods

### Participants

A survey was provided on web sites of relevant agencies in Virginia, including Early Intervention, Commonwealth Autism Service, Blue Ridge Autism Center, Autism Society of America – Greater Roanoke Valley Chapter, and the Virginia Tech Autism Clinic. Anyone who came across the survey was able to participate if they met the inclusion criteria (i.e., were a parent or caregiver of a person with ASD and currently resided in Virginia). In addition, administrators of the above-noted web sites sent email notices to their membership alerting caregivers to this survey. The email message directed caregivers to the agency's web site to self-initiate the survey. The final sample consisted of 146 respondents.

See Table 1 for Descriptive information about the sample. The sample consisted primarily of mothers (75%, n = 110) of individuals with ASDs. The majority of the respondents (88%, n = 129) were Caucasian/European American and most (56%, n = 82) reported having some college or a college degree, or education beyond college (32%, n = 46). Approximately equal proportions of respondents reported incomes less than $40,000 (24%, n = 35), between $40–59,999 (25%, n = 37), between $60–79,999 (18%, n = 27), and above $80,000 (24%, n = 35) annually. As part of the inclusion criteria, all respondents resided in Virginia. Most had been living in Virginia (79%, n = 110) and in their current county (65%, n = 92) for over a decade, with approximately a third of the sample currently residing in Montgomery County (29%, n = 38). Most respondents lived in mixed (rural and urban) counties (47%, n = 67), while 21% (n = 30) lived in rural and 32% (n = 46) in urban areas. The majority of children reported on were boys (83%, n = 121) and most had a diagnosis of Autism/Autistic Disorder (57%, n = 83). The mean age at the time of diagnosis was 58 months (4 years, 10 months; SD = 37.39), and the most frequent age of diagnosis was 36 months (3 years old).

**Table 1:** Demographic information

Relation to child	Number of participants (percentage)
Mother	110 (75%)
Father	15 (10%)
Step mother	3 (2%)
Legal guardian	2 (1%)
Adoptive mother	2 (1%)
Other	14 (10%)
Race/ethnicity	Number of participants (percentage)
African American	7 (5%)
Asian/Pacific Islander	3 (2%)
Caucasian/European American	129 (88%)
Native American	1 (1%)
Latino, Hispanic, or Chicano	1 (1%)
No answer	3 (2%)
Highest level of education	Number of participants (percentage)
Some high school	1 (1%)
High school graduate	15 (10%)
Some college	42 (29%)
College degree	40 (27%)
Some graduate studies	14 (10%)
Graduate degree	32 (22%)
No answer	2 (1%)
Annual Income	Number of participants (percentage)
Under $20,000	7 (5%)
$20,000–39,999	28 (19%)
$40,000–59,999	37 (25%)
$60,000–79,999	27 (18%)
$80,000–99,999	15 (10%)
$100,000 and above	20 (14%)
No answer	12 (8%)
Years lived in Virginia	Number of participants (percentage)
1–10	29 (21%)
11–20	23 (17%)
21–30	24 (17%)
31–40	39 (28%)
41–50	14 (10%)
51 or more	10 (7%)
Years in local area	Number of participants (percentage)
1–10	49 (35%)
11–20	45 (32%)
21–30	22 (16%)
31–40	19 (13%)
41–50	4 (3%)
51 or more	2 (1%)
Rural status	Number of participants (percentage)
Rural	30 (21%)
Mixed	67 (47%)
Urban	46 (32%)

### Design and Procedure

The survey consisted of three sections that were provided in the same order for all participants. If the respondent had more than one child with ASD, they were asked to respond to items in reference to the oldest child. Additional items were included in the survey, but are not detailed here because we had no specific hypotheses about them in relation to age of diagnosis or information provided by professionals (see Additional files 1).

The first section included general and demographic information (i.e., relationship to child, race/ethnicity, education, income, education, county of residence, etc.). County of residence was further coded as 2 = rural, 1 = mixed, and 0 = urban, according to Virginia 2006 status codes (based on population density). Demographic information is presented in Table 1.

The second section included questions specific to the child's ASD diagnosis (i.e., 3 = Autistic Disorder, 2 = Asperger's Disorder, 1 = Pervasive Developmental Disorder – Not Otherwise Specified), and age of initial diagnosis in months. Current ASD diagnosis was coded to reflect the spectrum from atypical to classic autism. This section also included questions related to the provider that initially diagnosed the child (i.e., 4 = Developmental Pediatrician, 3 = Psychologist, 2 = Neurologist, and 1 = Other/Psychiatrist/Primary Care Physician) and what information was provided by this diagnosing professional (i.e., Provided no additional information, Gave information about available resources, Gave literature about autism, Spent time talking about autism, Referred to an autism specialist, Referred to a support group, Advised on educational programs, Advised on medical programs). For analyses, the item on the provider was coded in order of increasing training that is specialized in early childhood development or ASD. That is, developmental pediatricians were considered to have the most early childhood/ASD training, and the others were considered to be about equal or varied in training. The item on information provided was coded as either 1 = 'no' (i.e., parent endorsed that the professional provided no additional information beyond the diagnosis) or 0 = 'yes' (i.e., if any of the listed forms of information were endorsed). Lastly, this section also asked respondents to report who/what helped them learn about ASD upon receiving the initial diagnosis (i.e., Healthcare Professionals, Education Professionals, Parent Resource Centers, Parents of children with Autism, Family member, Friends, Support Groups, Advocacy groups, Internet, Books, Magazines, and Videotapes, Conferences and Workshops, Did not seek further information, Other), and whether they were currently or previously in a parent support group or parent advocacy group for ASD.

The final section surveyed parents' opinions regarding the status of autism-related outpatient services in their local area. Parents were asked to identify which services they had used or were currently using and then evaluate the services using a scale from 1–5 with 5 being the highest. Respondents were presented with a range of potential biomedical, behavioral, and educational services. Ratings were based on the availability and ease of locating the service in their area (within a forty mile radius), their satisfaction with the quality of the services, and the perceived need for such services in their area.

This study was approved under Exempt status by the Human Subjects Committee of the Department of Psychology and the Institutional Review Board at Virginia Polytechnic Institute and University, in compliance with the Helsinki Declaration. A study description was provided online immediately preceding the survey. The description outlined the contents of the survey and stated that participation was completely voluntary, anonymous, and could be withdrawn at any time. Participants also were informed that they could skip questions, if desired. There was no compensation for participation. As permitted for Exempt status, submission of the survey served as informed consent.

## Results

### Age of diagnosis

As noted above, the reported mean age of diagnosis was 58 months (4 years, 10 months; SD = 37.39). The modal age of diagnosis was 36 months (3 years old) and the median was 45 months (3 years, 9 months). This suggests that most of the children were initially diagnosed in the pre-school years, as hypothesized, and 50% were older than 3 years, 9 months at the time of their initial diagnosis. To further evaluate what factors might be involved in an earlier age of diagnosis, a multiple regression analysis was conducted to examine the effect of demographic variables (including race, income, education level of parent, rural/mixed/urban region, and child's gender) as well as current diagnosis (i.e., Autism, Asperger's Disorder, PDD-NOS) and provider type on the age of diagnosis in months. Current diagnosis was assumed to reflect the level of the child's symptomatology along the ASD spectrum. Provider type (i.e., psychiatrist/primary care physician, neurologist, psychologist, or developmental pediatrician) was assumed to reflect the level of specialized early childhood/ASD training on the part of the diagnosing professional. See Table 2 for a presentation of the multiple regression findings. The overall regression model, with all variables entered simultaneously, was significant, *R*^2 ^= .133, *F*(7, 110) = 2.402, *p *= .025. The results indicated no significant demographic effects; however, the provider type and diagnosis were significant. Specifically, developmental pediatricians were associated with earlier ages of diagnosis,*Standardized Beta *= -.249, partial *t *= -2.73, *p *= .007. A oneway Analysis of Variance (ANOVA) confirmed this analysis, *F*(3,133) = 4.97, *p *= .003, with developmental pediatricians (M = 47.59 months, SD = 28.85) diagnosing about one year younger than psychologists (M = 59.52 months, SD = 35.29) and neurologists (M = 60.46 months, SD = 32.50), and about 2.5 years younger than other physicians (M = 79.65 months, SD = 51.65). In addition, children with current Autism were reportedly diagnosed at earlier ages than those with Asperger's or PDD-NOS diagnoses, *Standardized Beta *= -.219, partial *t *= -2.43, *p *= .017. Again, this was confirmed by a oneway ANOVA, *F*(2,138) = 13.94, *p *= .000, with Autism (M = 47.51 months, SD = 32.56) diagnosed earlier than Asperger's Disorder (M = 85.03 months, SD = 29.52) or PDD-NOS (M = 57.26 months, SD = 43.64).

**Table 2:** Multiple regression predicting age of diagnosis in months

	Unstandardized Coefficients		Standardized Coefficients		
	
	B	Std. Error	Beta	t	p-value
Rural status	5.951	4.743	.116	1.255	.212
Race	-.867	11.156	-.007	-.078	.938
Education	4.094	5.673	.069	.722	.472
Income	-1.255	2.487	-.050	-.505	.615
Gender	-.686	9.732	-.006	-.071	.944
Diagnosis	-10.102	4.163	-.219	-2.427	.017
Type of Professional	-7.766	2.839	-.249	-2.735	.007

### Information provided by diagnosing professional

See Table 3 for findings on the diagnosing professional and subsequent information provided to or obtained by parents. As can be seen, in total, the majority of professionals reported to diagnose the children were physicians, including developmental pediatricians, neurologists, primary care physicians, and psychiatrists (67%, n = 98). Of these various physicians, respondents most frequently reported that a developmental pediatrician initially diagnosed their child (43%, n = 63). According to parents, in approximately 40% of cases (41–45%, n = 60–66), the professional who made the diagnosis also provided additional information about autism and available resources and spent some time discussing autism with the family. In other cases (6–34%, n = 9–50), the professional referred the family to specialists, educational programs, or support groups. However, in 18% of reported cases (n = 26), the professional provided no additional information beyond the diagnosis

**Table 3:** Diagnostic information

Professional Diagnosing the Child	Number of participants (percentage)
Developmental pediatrician	63 (43%)
Psychologist	24 (16%)
Neurologist	25 (17%)
Primary care physician	1 (1%)
Psychiatrist	9 (6%)
Other	18 (12%)
No answer	6 (4%)
Information Provided After Diagnosing the Child	Number of participants (percentage)
Provided no additional information	26 (18%)
Gave information about available resources	66 (45%)
Gave literature about autism	65 (45%)
Spent time talking about autism	60 (41%)
Referred to an autism specialist	9 (6%)
Referred to a support group	14 (10%)
Advised on educational programs	50 (34%)
Advised on medical programs	22 (15%)
Other	21 (14%)
Who/what helped in learning about ASD	Number of participants (percentage)
Healthcare professionals	29 (20%)
Education professionals	31 (21%)
Parent resource centers	26 (18%)
Early intervention specialist	22 (15%)
Parents of children with Autism	61 (42%)
Family member	12 (8%)
Friends	15 (10%)
Support group	43 (29%)
Advocacy group	25 (17%)
Internet	107 (73%)
Books, magazines, or videotapes	103 (71%)
Conferences and workshops	61 (42%)
Didn't seek further help	0
Other	12 (8%)
Membership status of support groups	Number of participants (percentage)
Member of an autism specific group	69 (47%)
Member of a non-autism specific group	8 (5%)
Member of autism and non-autism specific group	17 (12%)
Not currently, but member in the past	11 (8%)
Never a member	18 (12%)
Not currently, but wants to be a member	21 (14%)
No answer	2 (1%)

To further evaluate what factors might be involved in the provision of information, multinomial logistic regression analysis was conducted to examine the effect of demographic variables (including race, income, education level of parent, rural/mixed/urban region, and child's gender) as well as current diagnosis (i.e., Autism, Asperger's Disorder, PDD-NOS) and provider type on whether or not the professional provided further information about ASD beyond the diagnosis. A logistic regression was used because the outcome variable was dichotomous. See Table 4 for a presentation of the logistic regression findings. The overall regression model, with all demographic variables entered first, was significant, *Chi-Square *= 28.87, *df *= 10, *p *= .001. The results indicated no significant demographic effects and no significant effect for current diagnosis; however, the provider type was significant. Specifically, developmental pediatricians were more likely than other professionals to provide additional information at the time of diagnosis, *Chi-Square *= 25.07, *df *= 3, *p *= .000. A Pearson Chi-square supported these findings, indicating that 96.8% of developmental pediatricians were reported to provide further information, compared to 77.8% of psychiatrists/primary care physicians, 75% of psychologists, and 56% of neurologists, *Chi-Square *= 21.97, *df *= 3, *p *= .000.

**Table 4:** Logistic regression predicting whether information was provided by the diagnosing professional

	Model Fitting Criteria	Likelihood Ratio Tests		
	
Effect	-2 Log Likelihood of Reduced Model	Chi-Square	Df	p-value.
Rural Status	87.012	2.207	1	.137
Race	84.826	.021	1	.884
Education	84.905	.100	1	.752
Income	86.980	2.175	1	.140
Gender	86.325	1.519	1	.218
Diagnosis	88.729	3.924	2	.141
Type of Professional	109.876	25.071	3	.000

### Sources of information

When asked what source helped parents learn more about ASD when the initial diagnosis was received, parents most often reported turning to the media (71–73%, n = 103–107; internet, books, videos, etc.), conferences/workshops (42%, n = 61), or other parents (42%, n = 61). Only 15–20% (n = 22–29) of parents reported obtaining this information from local healthcare, educational, or early intervention professionals. Most parents (64%, n = 94) did report belonging to a parent support or advocacy group. As such, the respondents were less likely to learn about ASD from local healthcare or educational professionals, and more likely to learn from media, conferences, and other parents. See Table 3 for percentages of respondents reporting each source of support.

See Table 5 for a summary of the perceived availability and need for autism-related services. Responses were grouped into those that were rated as high in need (rating of 3–5) and low in local availability (rating of 1–2). The top 5 services that were rated as both highly needed but with little availability included Behavioral Treatment, Social Skills Training, Autism Specialty Clinics, Diagnostic Services, and Sensory or Auditory Integration.

**Table 5:** Services perceived as high In need and low in availability

Service	Number of participants that rated high need (3–5)	Number of participants that rated low in availability (1–2)
Behavioral Treatment	103 (71%)	62 (42%)
Social Skills Training	103 (71%)	55 (38%)
Autism Specialty Clinics	97 (67%)	68 (46%)
Diagnostic Services	96 (66%)	45 (31%)
Sensory or Auditory Training	90 (61%)	42 (29%)

## Discussion

In this sample, the average age of diagnosis was 4 years, 10 months, but the most frequent was 3 years, consistent with our hypothesis that most children would be diagnosed between ages 3 and 4. Moreover, 50% of the sample was reportedly diagnosed after 3 years, 9 months. Developmental pediatricians were associated with the lowest average age of diagnosis (i.e., 4 years) and Autism was diagnosed earlier than Asperger's Disorder or PDD-NOS. Contrary to our prediction, the vast majority of professionals (82%) gave some type of additional information at the time of diagnosis. While approximately 40% of professionals gave additional information about autism and 15–34% offered advice on educational or medical programs, few professionals (6%) made referrals to autism specialists and 18% provided no additional information about ASD or related services. Developmental pediatricians were most likely reported to provide such additional information and referral. Finally, our hypothesis that the majority of caretakers would turn to sources of information about ASD outside of their healthcare or service provider was supported in that most of the respondents used the media (71–73%; internet, books, videos, etc.) and many turned to conferences/workshops (42%) or other parents (42%).

Although all children should receive needed services regardless of age, they have a much more positive developmental trajectory if interventions begin before age 5, and even greater gains if before age 3 [14-17]. Our finding that most children received their diagnosis at age 3 or later underscores the need for earlier diagnosis and the important role pediatricians can play in this regard. We found that children with Autism were diagnosed younger than children with other ASDs and developmental pediatricians were most likely to provide earlier diagnoses. These findings suggest that the presence of more "classic" (and arguably more identifiable) autism symptoms and specialized training in early childhood development may lead to younger ages of diagnosis. If this is the case, the implication is that increased training of professionals on early signs and on the range of characteristics associated with the broad autism spectrum would improve earlier detection and diagnosis.

Results of the present study suggest that although the majority of physicians are giving parents information about ASD, this avenue of support can be significantly strengthened. To help families, physicians must be knowledgeable of the current research on empirically supported treatments for ASD. Unfortunately, some physicians may have misconceptions about ASD [3] or are not adequately taught in medical school about details such as the causation, IQ profiles, prognosis, and treatments for ASD [13]. Such lack of knowledge could explain why some pediatricians hesitate to give information at the time of diagnosis. Our finding that developmental pediatricians were most likely to provide additional information to families (96.8%) than other health care professionals perhaps reflects their greater degree of specialized training in ASD. However, it should be noted that there may be other reasons not assessed here for this finding. For example, family or community resources may influence which type of professional a family sees.

Many respondents reported that they turned to the internet/media to get further information about ASDs. While these sources provide valuable information, the large volume can be overwhelming. As trusted sources of information, physicians are in a perfect role to guide families toward information with empirical support. Our finding that caregivers listed Behavioral Therapy, Social Skills Training, Autism Specialty Clinics, Diagnostic Services, and Sensory or Auditory Integration as both high in need and low in availability, suggest that they are likely to seek such services. While Behavioral Therapy [14-20] and Social Skills Training [21] have some empirical support, Sensory and Auditory Integration Training generally do not [22-24]. If physicians are not knowledgeable of treatment options or do not discuss this with caregivers, they may inadvertently encourage the use of treatments that lack empirical support. In addition, because we found that all of these services are in fact so low in local availability, physicians in this sample may indeed have had little to offer by way of advice or referral, even if knowledgeable about the best practices. Nonetheless, it would be important to provide information on evidence-based treatments and allow parents the opportunity to seek them out or lobby for their development in local areas.

Another way for parents to obtain information about ASDs is through support groups, and most caregivers (64%) in our sample did report being a member of a parent support or advocacy group. Such groups are not only an excellent way for caregivers to relieve stress, but also provide a means to relay information about local services. Again, however, it would be important for health care professionals to be involved in helping parents sort through the plethora of information they may receive.

Although not significant in our study, it is important to note prior findings that rural children (i.e., defined as living in a town of fewer than 2,500 people or as a non-metropolitan area) receive a diagnosis approximately 5 months later than urban children [25]. People living in rural areas may be less likely to be targeted by health-promotion/disease-prevention interventions [26]. We did not find that rural status significantly predicted either age of diagnosis or the information provided by the diagnosing professional. However, an examination of the means suggests otherwise. The average age of diagnosis was 64 months for rural areas, compared to 56 months for both urban and mixed areas (i.e., a lag of 8 months in rural areas). Moreover, 21% of rural residents and 23% of mixed area residents, but only 11% of urban residents, received no further information from their diagnostic provider. As such, although we caution that these values were not statistically significant, future research needs to further explore and tackle this important rural mental health issue.

## Conclusion

In sum, we found that 1) most children with ASD were diagnosed at age 3 or later, 2) a substantial minority of diagnosing professionals (18%) provided no further information about ASD, and 3) caregivers reported turning to the media, conferences, or other parents to learn more about ASD. We found that developmental pediatricians were most likely to diagnose at earlier ages and provide families with additional information, and that Autism was associated with earlier diagnoses than other ASDs.

Our findings suggest, but do not prove, that pediatricians with specialized training make earlier diagnoses and are more likely to provide additional information to caregivers. One implication to address this potential problem would be to have all pediatricians and health care professionals attend a brief training about the current state of knowledge on early signs and treatment of ASD, similar to that described in Koegal et al. [27]. This may be difficult to fund and replicate across the country, but would be worthwhile by arming physicians with training to feel confident in making early referrals/diagnoses and in advising caregivers on evidence-based practices.

There may be multiple reasons for a delay in diagnosis (e.g., long wait-lists for specialists, lack of local specialists, delay in follow-through after a referral is made), but one potential problem could be delays in detection. Therefore, another implication is the physician's ability to make earlier referrals and/or diagnoses by using reliable ASD screening and diagnostic tools, such as the Checklist for Autism in Toddlers (completed by physicians), a modified version called the M-CHAT (completed by parents), the Developmental Behavior Checklist, and the Social Communication Questionnaire [28-30]. After screening, children with suspected ASD should be referred for diagnostic testing using evidence-based assessments [31] that include clinical interviews of the parent (e.g., the Autism Diagnostic Interview – Revised) and observations of the child (e.g., the Autism Diagnostic Observation Schedule).

There are several limitations of this study. First, the sampling strategy could have introduced a bias from the websites on which the survey was posted. This could be viewed as a convenience sample and may affect the results by some process of self-selection. For example, it is possible that caregivers who were disappointed in their provider care or local services may have been more likely to respond to this survey. However, because the websites were autism-related, we felt that this was the best way to reach caregivers of children with ASDs. A second limitation is the small sample size, which may limit ability to generalize the results to a larger population. A third limitation is that we did not include survey questions on the diagnosing professional's level of ASD training, when this training was, or the professional's extent of contact with children with ASD. Although we categorized professionals according to their presumed level of specialized training, we did not specifically inquire about this training. Further research in the area should address these issues in order to clarify factors that may impact the response of the professional.

In conclusion, the late age of diagnosis and lack of information given about ASD could be due to multiple factors not assessed here, including limited time spent with patients [27], limited training [13], reluctance to diagnose because of emotional reactions [10], lack of health care in general [26], or resource issues that impede families from obtaining early help. Training in the broad autism spectrum and its early development may improve detection in physicians who see young children. Efforts should be made to alleviate such problems so that children with ASDs can receive intervention as early as possible and thus have improved developmental trajectories.

## Competing interests

The author(s) declare that they have no competing interests.

## Authors' contributions

RAR compiled the data from the survey, conducted initial analyses, and wrote the first draft of the text. AS conceived of and designed the study, was in charge of creating the survey, conducted supplementary analyses, edited and revised the text. BS edited the initial draft and helped with initial revisions of the text. All authors read and approved the final manuscript.

## Pre-publication history

The pre-publication history for this paper can be accessed here:



## Supplementary Material

Additional File 1 Survey: assessing the needs of individuals with autism spectrum disorder in Virginia. Copy of the parent/caregiver survey from the study about the needs of children with autism spectrum disorder. Click here for file
