# Supplementary material for: The importance of physician knowledge of autism spectrum disorder: results of a parent survey
Source: BMC Pediatr. 2007 Nov 20;7:37. doi: 10.1186/1471-2431-7-37 (PMC2235850; doi:10.1186/1471-2431-7-37)
Supplement: Additional File 1 — Survey: assessing the needs of individuals with autism spectrum disorder in Virginia. Copy of the parent/caregiver survey from the study about the needs of children with autism spectrum disorder. [file 1471-2431-7-37-S1.doc]

**Survey: Assessing the Needs of Individuals with Autism Spectrum Disorder in Virginia**

Dear family member of a person with Autism Spectrum Disorder:

We greatly appreciate your help by filling out this survey. This survey is being collected by the Virginia Tech Autism Research Group and the NRV Autism Action Group with the purpose of evaluating the service needs for those diagnosed with Autism Spectrum Disorder in Virginia.

Please answer as many questions as you can. Since most people completing the survey will be parents, we call the individual with Autism Spectrum Disorder “your child” throughout the survey, though we understand your “child” may now be an adolescent or adult. If you have more than one child with Autism Spectrum Disorder, please complete the survey for the oldest child.

Autism Spectrum Disorders are classified as pervasive developmental disorders (PDDs) and include: Autism, Childhood Disintegrative Disorder, Rett’s Syndrome, Asperger’s Syndrome, and Pervasive Developmental Disorder – Not Otherwise Specified (PDD-NOS).

Your participation is completely voluntary. Your responses are anonymous, meaning your name does not appear on this survey and is not associated with any responses. You do not have to answer any questions that you do not want to answer. If you have any questions about the survey please email our director, Dr. Angela Scarpa, at [ascarpa@vt.edu](mailto:ascarpa@vt.edu).

Returning the completed survey will serve as your consent to participate. **Please return the survey in the enclosed pre-addressed stamped envelope.**

If you would like us to send you a summary of the findings and include you in future surveys or research on Autism Spectrum Disorder, please write your name and address on the enclosed pre-addressed stamped postcard. We will keep your name on file and send you a summary of our findings upon completion of the study.

Thank you again for your time and effort. The information you provide is critical to improving services for people with Autism Spectrum Disorder in the New River Valley.

Sincerely,

Virginia Tech Autism Research Group

NRV Autism Action Group

**General Information About the Parent**

1. What relation are you to the child?

___ Mother

___ Father

___ Step Mother

___ Step Father

___ Adoptive Mother

___ Adoptive Father

___ Legal Guardian

___ Other Relative (please specify___________________________)

1. What is your race/ethnicity? (optional)

___ African American

___ Asian/Pacific Islander

___ Native Hawaiian

___ Caucasian/European American

___ Native American

___ Latino, Hispanic, or Chicano

___ Other (please specify __________________________________)

1. What is your highest level of completed education? (optional)

___ Some High School

___ High School graduate

___ Some college

___ College degree

___ Some graduate studies

___ Graduate degree

1. Which of the following is closest to your annual household income? (optional)

___ Under $20,000

___ $20,000 - $39,999

___ $40,000 - $59,999

___ $60,000 – $79,999

___ $80,000 - $99,999

___ $100,000 and above

1. How long have you lived in Virginia?

___ years

___ months

Please list all the counties where you have resided in Virginia: _____________________________________________________________________

1. In what county do you currently reside? ­­­­­­­­­­­­­­­___________________________________

**General Information About Child**

1. How old is your child?

­­­­­­­­­­­_________ years

_________ months

1. What is your child’s gender?

___ Male

___ Female

1. What level of education has your child completed?

___ None

___ Preschool

___ Kindergarten

___ Elementary School (specify highest grade completed ____)

___ Middle School (specify highest grade completed ____)

___ High School (specify highest grade completed____)

___ College

___ Graduate School

___ Home schooling (specify highest grade completed ____)

**General Information on Diagnosis and Professional Services**

1. What is your child’s current diagnosis?

___ Autism Disorder

___ Asperger’s Disorder

___ Childhood Disintegrative Disorder

___ Rett’s Syndrome

___ Pervasive Developmental Disorder – Not Otherwise Specified (PDD-NOS)

___ Other (please specify __________________________)

1. How old was your child when he/she got the diagnosis?

___ years

___ months

1. Have any of the child’s siblings been diagnosed with Autism Spectrum Disorder?

___ yes (if yes, please specify which disorder:­­­­­­­­­­­­­­_________________________)

___ no

1. Does your child have other symptoms or diagnoses? (check all that apply)

___ Anxiety Disorder

___ Attention Deficit Hyperactive Disorder (ADHD)

___ Obsessive Compulsive Disorder

___ Central Auditory Processing Disorder

___ Depression

___ Schizophrenia

___ Hearing Impairment

___ Vision Impairment

___ ‘Tunnel Vision Syndrome’ (peripheral vision, vision perception impairment)

___ Mental Retardation

___ Seizures

___ Dietary allergies (please specify ____________________________)

___ Digestive Problems (constipation, diarrhea, bloating, or abdominal pain)

___ None

___ Other (please specify _____________________________________)

1. What type of professional diagnosed your child with Autism Spectrum Disorder?

___ Developmental Pediatrician

___ Psychologist

___ Neurologist

___ Primary Care Physician

___ Psychiatrist

___ Other (please specify _____________________________________)

1. What did that professional do after diagnosing your child? Check all that apply.

___ Provided no additional information

___ Gave you information about available resources

___ Gave you literature on Autism

___ Spent time talking with you about Autism

___ Referred you to an Autism specialist

___ Referred you to a support group

___ Advised on educational programs

___ Advised on medical problems

___ Other (please specify _____________________________________)

1. When you first received the diagnosis, who/what helped you to learn about Autism Spectrum Disorder?

___ Healthcare Professionals

___ Education Professionals

___ Parent Resource Centers

___ Parents of children with Autism

___ Family member

___ Friends

___ Support Groups

___ Advocacy groups

___ Internet

___ Books, Magazines, and Videotapes

___ Conferences and Workshops

___ I did not seek further information

___ Other (please specify ____________________________________________)

1. Are you a member of a support group or advocacy group for parents?

___ Yes, autism specific

___ Yes, not autism specific

___ Yes, both autism specific and not autism specific

___ No, but was a member in the past

___ No, have never been a member

___ No, but I want to belong to a group.

1. Have you ever moved to another school district to get better services for your child?

___ yes

___ no

**Current Symptoms Displayed by Child**

Please check the symptoms currently exhibited by your child: (check all that apply)

___ Doesn’t respond when called

___ Self-injurious behaviors

___ Destructive behaviors

___ Receptive language delay

___ Expressive language delay

___ No verbal language

___ Apraxia (oral motor, articulation problems)

___ Absent or limited gestures

___ Cognitive delay

___ Strong visual learner

___ Strong auditory learner

___ Gross motor delay

___ Fine motor delay

___ Undersensitive to pain

___ Oversensitive to pain

___ Undersensitive to sound

___ Oversensitive to sound

___ Aggressive to others

___ Has trouble joining a group

___ Happier left alone

___ Frustrated

___ Gets angry easily

___ Cries excessively

___ Hums frequently

___ Insists on sameness

___ Agitated when routine is disrupted

___ Insists on precision

___ Poor eye contact

___ Stomach pain

___ Constipation

___ Diarrhea

___ Eczema

___ Thrush (white tongue yeast infection)

___ Itchy penis/perineum/all

___ Losing weight

___ Gaining weight

___ Fixation on objects or topics

___ Unusual cravings for certain foods (please specify: _______________________)

___ Has known food sensitivity (please specify: _______________________)

___ Sustained odd play

___ Echolalia (repeats the same phrase over and over)

___ Does not require long sleep

___ Requires longer than average sleep

___ Does not stay asleep

___ Wakes up at night and does not go back to sleep

___ Takes a long nap daily

___ Tantrums

___ Anxiety

___ Depression

___ Hand flapping

___ Toe walking

___ Spinning Self

___ Likes to watch object spin

___ Rhythmic or rocking behaviors

___ Other types of self-stimulatory behavior (please specify: ______________________)

*This is a list of general services that could be available for children with Autism Spectrum Disorder.*

*Of these services, place a checkmark in the corresponding column for those you currently use or have used*.

*Then,* ***whether or not you have used these services****, please rate your opinion of the following:*

*1. First, please rate your opinion of the degree to which these services are currently available in Virginia (within a 40 mile radius of your residence).*

*2. Second, please rate your opinion of the general quality of those services. If not available in Virginia, circle N/A.*

*3. Third, please rate your opinion of the need/importance for these services to be offered in Virginia.*

*Circle the number that best reflects your opinion, using the following scale.*

**Very Low Low Moderate High Very High**

***1 2 3 4 5***

|  | **USING** | **USED IN PAST** | **AVAILABILITY** | **QUALITY** | **NEED** |
| --- | --- | --- | --- | --- | --- |
| **BEHAVIORAL TREATMENT** (such as Applied Behavior Analysis, Pivotal Response Training, Discrete Trial Training, or Positive Behavior Supports) |  |  | 1 2 3 4 5 | 1 2 3 4 5 N/A | 1 2 3 4 5 |
| **SOCIAL SKILLS TRAINING** |  |  | 1 2 3 4 5 | 1 2 3 4 5 N/A | 1 2 3 4 5 |
| **SPEECH-LANGUAGE THERAPY** |  |  | 1 2 3 4 5 | 1 2 3 4 5 N/A | 1 2 3 4 5 |
| **EARLY INTERVENTION (0-3 YEARS)** |  |  | 1 2 3 4 5 | 1 2 3 4 5 N/A | 1 2 3 4 5 |
| **OCCUPATIONAL THERAPY** |  |  | 1 2 3 4 5 | 1 2 3 4 5 N/A | 1 2 3 4 5 |
| **PLAY THERAPY** |  |  | 1 2 3 4 5 | 1 2 3 4 5 N/A | 1 2 3 4 5 |
| **FLOORTIME/GREENSPAN** |  |  | 1 2 3 4 5 | 1 2 3 4 5 N/A | 1 2 3 4 5 |
| **MUSIC, DANCE, OR DRAMA THERAPY** |  |  | 1 2 3 4 5 | 1 2 3 4 5 N/A | 1 2 3 4 5 |
| **SENSORY INTEGRATION THERAPY** |  |  | 1 2 3 4 5 | 1 2 3 4 5 N/A | 1 2 3 4 5 |
| **PHYSICAL THERAPY** |  |  | 1 2 3 4 5 | 1 2 3 4 5 N/A | 1 2 3 4 5 |
| **VOCATIONAL TRAINING** |  |  | 1 2 3 4 5 | 1 2 3 4 5 N/A | 1 2 3 4 5 |
|  | **USING** | **USED IN PAST** | **AVAILABILITY** | **QUALITY** | **NEED** |
| **SOCIAL SKILLS TRAINING** |  |  | 1 2 3 4 5 | 1 2 3 4 5 N/A | 1 2 3 4 5 |
| **ANIMAL THERAPY** |  |  | 1 2 3 4 5 | 1 2 3 4 5 N/A | 1 2 3 4 5 |
| **DIETARY INTERVENTIONS** (such as Casein-free/Gluten-free diets, Fatty acids, Feingold diet, Alkaline shots, Magnesium, or Vitamin supplements) |  |  | 1 2 3 4 5 | 1 2 3 4 5 N/A | 1 2 3 4 5 |
| **RESPITE CARE** |  |  | 1 2 3 4 5 | 1 2 3 4 5 N/A | 1 2 3 4 5 |
| **FAMILY SERVICES** (such as Family Counseling, Parent Support Group, Sibling Support Group, Parent Advocacy Group, or Parent Training) |  |  | 1 2 3 4 5 | 1 2 3 4 5 N/A | 1 2 3 4 5 |
| **AUTISM SPECIALTY CLINICS/CENTERS** |  |  | 1 2 3 4 5 | 1 2 3 4 5 N/A | 1 2 3 4 5 |
| **MEDICATIONS** (such as SSRIs, stimulants, or antipsychotics) |  |  | 1 2 3 4 5 | 1 2 3 4 5 N/A | 1 2 3 4 5 |
| **DIAGNOSTIC SERVICES** |  |  | 1 2 3 4 5 | 1 2 3 4 5 N/A | 1 2 3 4 5 |
| **EDUCATOR'S TRAINING** |  |  | 1 2 3 4 5 | 1 2 3 4 5 N/A | 1 2 3 4 5 |
| **WEB-BASED RESOURCE NETWORK** |  |  | 1 2 3 4 5 | 1 2 3 4 5 N/A | 1 2 3 4 5 |
| **SPECIAL NEEDS CAMPS** |  |  | 1 2 3 4 5 | 1 2 3 4 5 N/A | 1 2 3 4 5 |

IN THE SPACE BELOW, PLEASE LIST THE MOST NEEDED SERVICES RELATED TO AUTISM SPECTRUM DISORDER IN VIRGINIA. YOU MAY CHOOSE FROM THE ABOVE LIST AS WELL AS ADD OTHERS THAT ARE NOT LISTED:

*-Please return this survey in the enclosed pre-addressed stamped envelope.*

*-If you would like us to send you a summary of the findings, please print your name and address on the enclosed pre-addressed stamped postcard and mail it in separately from this survey.*

-Thank you for completing this survey. Your responses will be extremely helpful in guiding appropriate services for affected people in Virginia.
